# Expression and prognostic significance of TRPV6 in the development and progression of pancreatic cancer

**DOI:** 10.3892/or.2022.8395

**Published:** 2022-09-05

**Authors:** He Song, Ming Dong, Jianping Zhou, Weiwei Sheng, Xin Li, Wei Gao

Oncol Rep 39: 1432–1440, 2018; DOI: 10.3892/or.2018.6216

Subsequently to the publication of the above article, an interested reader drew to the authors’ attention that Fig. 9A and C on p. 1438 contained some apparent errors in terms of the assembly of the various data panels. Specifically, two separate pairs of the data panels in Fig. 9 (namely, the panels showing the Migration/Negative control and Invasion/Negative control experiments for the SW1990 cell line in Fig. 9A, and those showing the Migration/TRPV6-Si and Invasion/TRPV6-Si experiments with the Capan-2 cell line in Fig. 9C) appeared to contain overlapping sections, such that they were derived from the same original sources. The authors have re-examined their raw data, and realized that some inadvertent errors were made during the compilation of this figure owing to mishandling of the data files.

The revised version of Fig. 9 (showing the data from one of the repeated experiments) is shown on the next page. The authors regret the errors that were made during the preparation of the published figures, but are able to confirm that these errors did not grossly affect the conclusions reported in the study. The authors are grateful to the Editor of *Oncology Reports* for allowing them the opportunity to publish a Corrigendum, and all the authors agree to this Corrigendum. Furthermore, they apologize to the readership for any inconvenience caused.

## Figures and Tables

**Figure 9.**
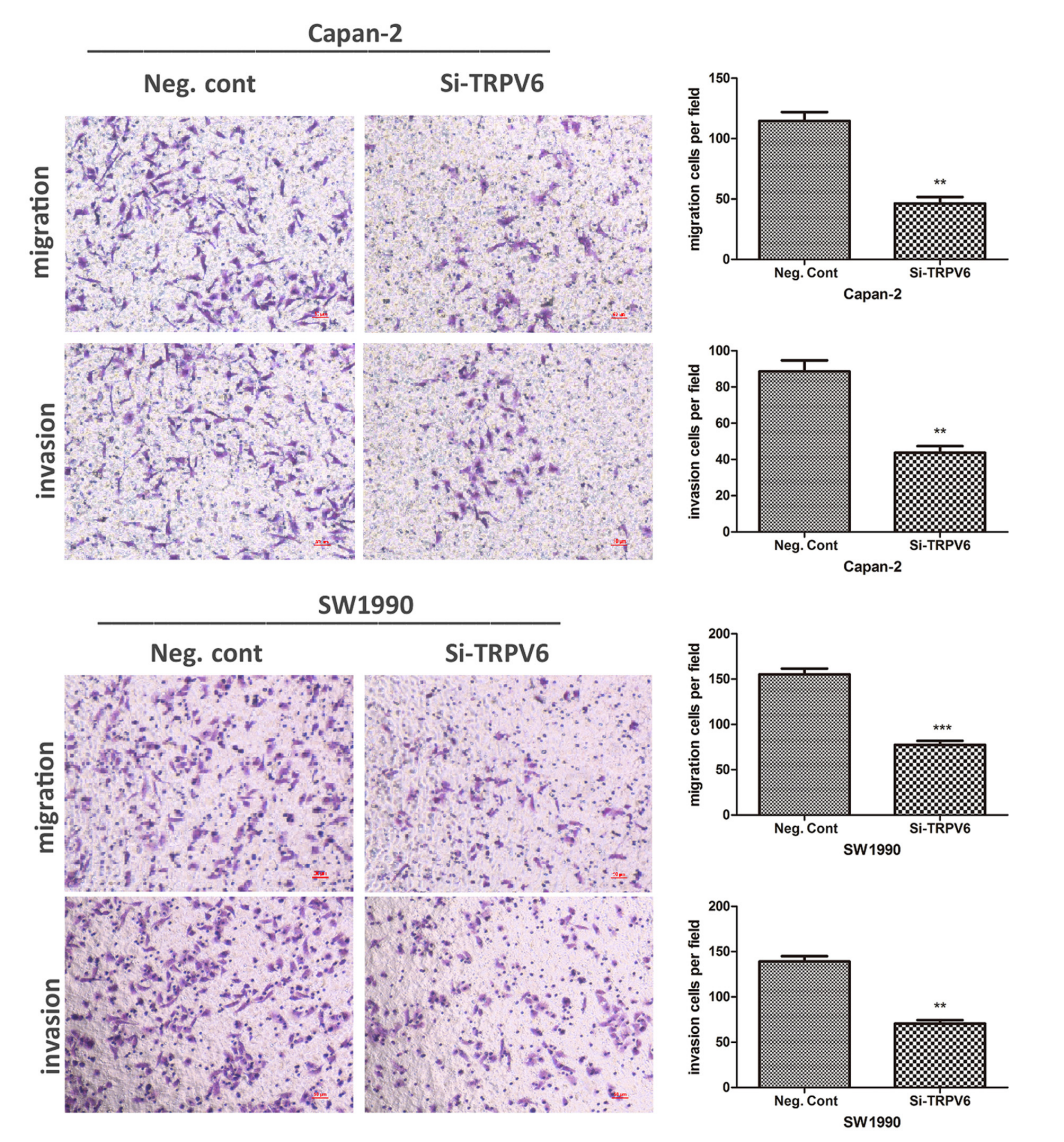
Downregulation of TRPV6 inhibits cell invasion and migration in the Capan-2 and SW1990 cell lines (A and C). Invasion and migration profiles of Capan-2 and SW1990 cells are depicted by histograms (B and D). **P<0.01, ***P<0.001.

